# JAK-inhibitor and type I interferon ability to produce favorable clinical outcomes in COVID-19 patients: a systematic review and meta-analysis

**DOI:** 10.1186/s12879-020-05730-z

**Published:** 2021-01-11

**Authors:** Lucas Walz, Avi J. Cohen, Andre P. Rebaza, James Vanchieri, Martin D. Slade, Charles S. Dela Cruz, Lokesh Sharma

**Affiliations:** 1 Department of Epidemiology of Microbial Diseases, Yale School of Public Health, New Haven, CT 06520 USA; 2 Section of Pulmonary and Critical Care and Sleep Medicine, Department of Internal Medicine, Yale University School of Medicine, S440, 300 Cedar Street, New Haven, CT 06520 USA; 3 Section of Pediatric Pulmonary, Allergy, Immunology and Sleep Medicine, Department of Pediatrics, Yale School of Medicine, New Haven, CT 06520 USA; 4 Department of Internal Medicine, Yale School of Medicine, New Haven, CT 06520 USA; 5 Department of Microbial Pathogenesis, Yale School of Medicine, New Haven, CT 06520 USA

**Keywords:** Viral infection, Respiratory infection, Infection control

## Abstract

**Background:**

The spread of a highly pathogenic, novel coronavirus (SARS-CoV-2) has emerged as a once-in-a-century pandemic, having already infected over 63 million people worldwide. Novel therapies are urgently needed. Janus kinase-inhibitors and Type I interferons have emerged as potential antiviral candidates for COVID-19 patients due to their proven efficacy against diseases with excessive cytokine release and their direct antiviral ability against viruses including coronaviruses, respectively.

**Methods:**

A search of MEDLINE and MedRxiv was conducted by three investigators from inception until July 30th 2020 and included any study type that compared treatment outcomes of humans treated with Janus kinase-inhibitor or Type I interferon against controls. Inclusion necessitated data with clearly indicated risk estimates or those that permitted their back-calculation. Outcomes were synthesized using RevMan.

**Results:**

Of 733 searched studies, we included four randomized and eleven non-randomized trials. Five of the studies were unpublished. Those who received Janus kinase-inhibitor had significantly reduced odds of mortality (OR, 0.12; 95% CI, 0.03–0.39, *p*< 0.001) and ICU admission (OR, 0.05; 95% CI, 0.01–0.26, p< 0.001), and had significantly increased odds of hospital discharge (OR, 22.76; 95% CI, 10.68–48.54, *p*< 0.00001) when compared to standard treatment group. Type I interferon recipients had significantly reduced odds of mortality (OR, 0.19; 95% CI, 0.04–0.85, *p*< 0.05), and increased odds of discharge bordering significance (OR, 1.89; 95% CI, 1.00–3.59, *p*=0.05).

**Conclusions:**

Janus kinase-inhibitor treatment is significantly associated with positive clinical outcomes in terms of mortality, ICU admission, and discharge. Type I interferon treatment is associated with positive clinical outcomes in regard to mortality and discharge. While these data show promise, additional well-conducted RCTs are needed to further elucidate the relationship between clinical outcomes and Janus kinase-inhibitors and Type I interferons in COVID-19 patients.

**Supplementary Information:**

The online version contains supplementary material available at 10.1186/s12879-020-05730-z.

## Background

The spread of a highly pathogenic novel coronavirus (SARS-CoV-2) has emerged as the deadliest pandemic since influenza in 1918, posing significant challenges for public health organizations, health care providers, and governments at all levels [1]. Severe disease caused by SARS-CoV-2 (COVID-19) has strained resources critical for patient care as well as those needed for protection of healthcare professionals such as personal protective equipment (PPE) around the world [2]. This, combined with limited therapeutic options, have led to Intensive Care Unit (ICU) mortality rates as high as 20% in some population subsets [3]. As of December 1st, SARS-CoV-2 has infected over 63 million people worldwide and led to the death of over 1.4 million patients [4]. Currently, only a few therapeutic approaches have been suggested to improve disease outcomes in susceptible populations, with only a few large-scale randomized clinical trials (RCTs) or observational studies having been conducted so far. These studies demonstrated modest effectiveness for agents such as remdesivir or dexamethasone [5, 6]. Additional therapeutics against COVID-19 are being explored, but in absence of large-scale RCTs, it remains difficult to assess the effectiveness of many of these therapies.

Janus-kinases (JAKs) are transmembrane proteins that mediate and amplify extracellular signals from growth factors and cytokines. Their inhibitors have been found to be effective in treating patients with inflammatory diseases [7]. These inhibitors function by targeting specific JAKs. Both Baricitinib and Ruxolitinib predominantly inhibit JAK1 and JAK2 [7]. JAK-inhibitors may be used to control high levels of cytokines and inflammation [8], as seen in patients with severe SARS-CoV-2 infection [9]. These inhibitors have proved helpful in “off-label” indications, where excessive cytokine release plays a central role in disease progression [10]. While the hypothesis of JAK-inhibitors successfully combating high levels of cytokine expression in SARS-CoV-2 infection has been reinforced in some small studies [11], their effect on a larger population has not been investigated.

Interferons, including Type I interferons (interferon-α/β) and Type III interferons (λ), are proteins secreted by infected cells that induce antiviral states in neighboring cells and stimulate cytokine production [12]. These interferons work through activation of JAK/STAT pathway to activate a multitude of genes that are collectively known as interferon-stimulated genes (ISGs). These ISGs act together to block the viral life cycle at different stages. Given the widespread expression of Type I interferon receptors, they function as broad-spectrum antivirals that can directly and indirectly inhibit the replication of by promoting the expression of ISGs [13]. These interferons have been found to have positive therapeutic effects in the treatment of viral infections including hepatitis [14], and coronaviruses such as SARS and MERS [15, 16]. One investigation assessed Type I recombinant interferon to be one of the most potent anti-SARS-CoV-2 antiviral agents [17]. Additionally, a recent investigation revealed that several severe cases of COVID-19 presented with a rare, X-chromosome loss-of-function mutation that impaired Type I interferon response [18], while another demonstrated an association between COVID-19 severity and Type I interferon deficiency [19]. Other studies have also implicated low levels of Type I and III interferons as partially responsible for the unique and inappropriate inflammatory response seen in COVID-19 patients [20]. Various studies have found reasons to support the use of Type I interferons in combination with other antivirals to promote positive outcomes among patients with COVID-19, but many are restricted by the limited number of patients treated with interferon [21]. Interestingly, these interferons perform their functions by activating the JAK pathway.

Uncertainty and a lack of clinically proven prophylactic and therapeutic options have precipitated the periodic update of treatment guidelines for patients infected with COVID-19. As such, systematic reviews evaluating effects in larger patient populations are necessary to ascertain drug-related COVID-19 outcomes. In this meta-analysis, we evaluate JAK-inhibitors and Type I interferons for their efficacy and ability to produce positive outcomes in patients infected with SARS-CoV-2.

## Methods

This systematic review was conducted in accordance with Preferred Reporting Items for Systematic Reviews and Meta-Analyses (PRISMA; Supplementary Table 5) [22].

### Search strategy and study quality assessment

MEDLINE (via PubMed) and MedRxiv were searched since inception throughout July 30th, 2020 by three investigators (LW, AC, JV). The following terms were searched in free-text fields for JAK-inhibitors. For MEDLINE: “COVID-19” AND “JAK inhibitor” OR “Ruxolitinib” OR “Tofacitinib” OR “Fedratinib” OR “Baricitinib” OR “Pacritinib”. For MedRxiv: “COVID-19 JAK inhibitor” OR “COVID-19 Ruxolitinib” OR “COVID-19 Tofacitinib” OR “COVID-19 Fedratinib” OR “COVID-19 Baricitinib” OR “COVID-19 Pacritinib”. The following terms were searched in free-text fields for Type I interferons. For MEDLINE: “COVID-19”[Title] AND “interferon”[Title/Abstract] OR “IFN”[Title/Abstract]. For MedRxiv: “COVID-19 interferon” or “COVID-19 IFN”.

Three investigators (LW, AJC, JV) independently screened titles and abstracts generated by the search. After selection, full electronic articles were then carefully evaluated for data extraction. Randomized studies included in the final analyses were scored by one investigator (LW) to formally assess for risk of bias utilizing the Risk of Bias (RoB) 2 tool (Supplementary Table 3) [23]. Non-randomized studies included in the final analyses were scored by one investigator (LW), utilizing the Newcastle-Ottawa Scale (NOS) according to the following study characteristics: (1) representativeness of exposed cohort, (2) selection of nonexposed cohort, (3) exposure assessment, (4) outcome of interest not present at the start of the study, (5) comparability of cohorts, (6) outcome assessment, (7) adequacy of length of time before follow-up, and (8) adequacy of follow-up of cohorts (Supplementary Table 4) [24].

### Inclusion and exclusion criteria

We included clinical trials that utilized combination or sole JAK-inhibitor or Type I interferon (IFN-α, IFN-β) for the treatment of confirmed COVID-19 infection. For inclusion, possible studies must have compared treatment outcomes of those treated with a JAK-inhibitor or Type I interferon against a defined control group that did not receive this treatment. Selection required data with clearly indicated risk ratios or odds ratios (OR), or those that permitted their back-calculation. Inclusion necessitated that the trial be a human study accessible in English, and could include pediatric or adult studies, observational studies, retrospective cohorts, randomized clinical trials, and case reports.

Studies that utilized in vivo or animal studies, as well as those examining histological, pathological, and cellular mechanisms were excluded. Duplicate studies, review articles, commentaries, and proposed protocols were also excluded. Trials were excluded if they primarily examined other therapies where outcomes were unclear as to which participants received JAK-inhibitors or Type I interferons. Finally, studies were not included if they presented outcomes considered heterogeneous across the review that made statistical synthesis impossible (e.g. Mean vs Median).

### Data extraction and data analysis

Each full article that met inclusion criteria was carefully reviewed with the following baseline information extracted: first author, publication year, country, study type, type of JAK-inhibitor or interferon used, number of total participants, number of participants receiving JAK-inhibitor or interferon, and outcome measurements (Table 1). The outcome measurements consolidated included mortality, disease severity (mild/moderate vs severe/critical), mechanical ventilation, ICU admission, discharge, and acute respiratory distress syndrome (Supplementary Table 1). Additional individual study definitions of COVID-19 disease severity are presented in Supplementary Table 2.

**Table 1:** Baseline characteristics of included studies. Included studies classifications of First Author, Year, Country, Study Type, Type of JAK-inhibitor/Type I interferon Used, Total Number of Participants, Number of Participants Receiving JAK-inhibitor/Type I interferon Used. Studies presented in alphabetical order by treatment group

First Author, Year	Country	Study Type	JAK-inhibitor/Interferon Used	Total # of Participants	N Participants Receiving JAK-inhibitor/Interferon^a^
Bronte 2020 [25]	Italy	Observational	Baricitinib	76	20
Cantini 2020a [26]	Italy	Retrospective Cohort	Baricitinib	191	113
Cantini 2020b [27]	Italy	Prospective Cohort, open-label	Baricitinib	24	12
Cao 2020 [28]	China	RCT	Ruxolitinib	41	20
Giudice 2020 [29]	Italy	RCT	Ruxolitinib	17	7
Chen 2020 [30]	China	Observational	IFN-alpha-2b	291	132
Davoudi-Monfared 2020 [31]	Iran	RCT	IFN-beta-1a	81	42
Du 2020 [32]	China	Retrospective Cohort	IFN-alpha	182	178
Estébanez 2020 [33]	Spain	Retrospective Cohort	IFN-beta-1b	256	106
Fan 2020 [34]	China	Retrospective Observational	IFN-alpha-1b	53	19
Hung 2020 [35]	China	RCT	IFN-beta-1b	127	86
Liu 2020 [36]	China	Retrospective Observational	IFN-alpha-2b	10	9
Pereda 2020 [37]	Cuba	Retrospective Cohort	IFN-alpha-2b	814	761
Wang 2020 [38]	China	Retrospective Cohort	IFN-alpha-2b	446	242
Zhou 2020 [39]	China	Retrospective Cohort	IFN^b^	221	139

*IFN* Interferon

^a^ No Study participants received JAK-inhibitor and Type I interferon

^b^ Unclear – Used in combination with Arbidol

ORs were extracted from articles or calculated from the presented data. Data were analyzed using Review Manager version 5.4 (Cochrane Corporation, Oxford, United Kingdom) and the Mantel-Haenszel method. All analyzed variables are dichotomous. Thus, crude ORs and 95% confidence intervals (CIs) are reported. Heterogeneity was assessed using tau-squared and chi-squared tests for random effects and fixed effect models, respectively, as well as the I^2^ statistic. For I^2^ > 50%, the random effects model was used. Otherwise, the fixed effects model was utilized. An alpha of 0.05 was adopted to determine significance.

## Results

**Fig. 1:**
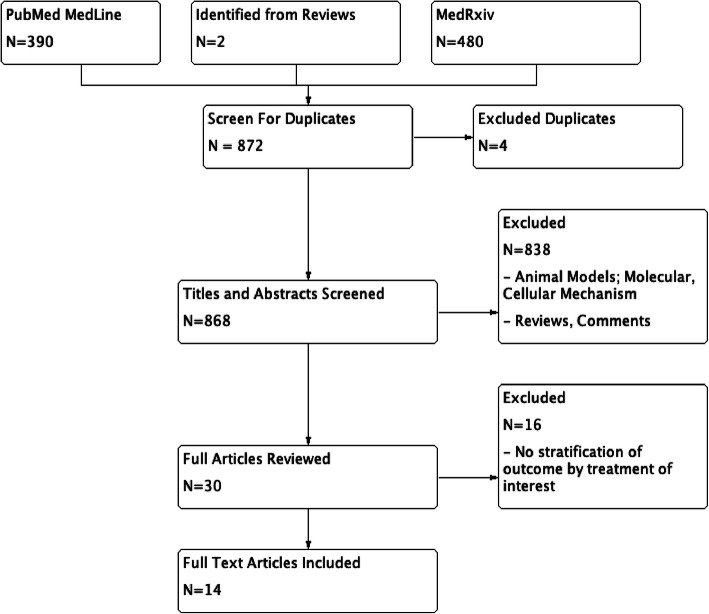
Flow diagram of study identification and assessment for eligibility. Two hundred twenty seven and 504 studied were identified from the databases Medline and MedRxIV, respectively. Two additional articles were added by manually searching retrieved reviews. Two articles were removed as duplicates. Six hundred ninety eight were removed after title and abstract screening not meeting inclusion criteria. Eighteen articles were removed after evaluation of the full article, with 15 included articles remaining

The initial database search returned 731 articles. Two additional articles were added by manually searching retrieved reviews. After removing two duplicates, 698 articles were excluded following title and abstract screening by three investigators. After comprehensive evaluation of 33 full-text articles, only 15 studies complied with the inclusion criteria. The majority of the studies excluded in the final step were excluded on the basis of not presenting outcome data in terms of those who did and did not receive JAK-inhibitor or interferon treatment. The remainder were excluded due to a focus on JAK inhibition as prophylaxis, a focus on interferon therapy as prophylaxis, or heterogeneity in reporting time among outcomes that precluded calculating pooled measures. Of the included studies, five were pre-prints. Overall, the 15 studies were comprised of four observational studies, six retrospective cohorts, four RCTs, and one prospective cohort. Figure 1 presents the meta-analysis flow chart and Table 1 presents the designs and characteristics of the included studies.

While some studies did not report which drugs were given as standard of care, many others reported treating patients with glucocorticoids, hydroxychloroquine, chloroquine, arbidol, and lopinavir/ritonavir. All studies were conducted within a hospital setting.

### Effect of JAK inhibition on clinical outcomes in COVID-19

**Fig. 2:**
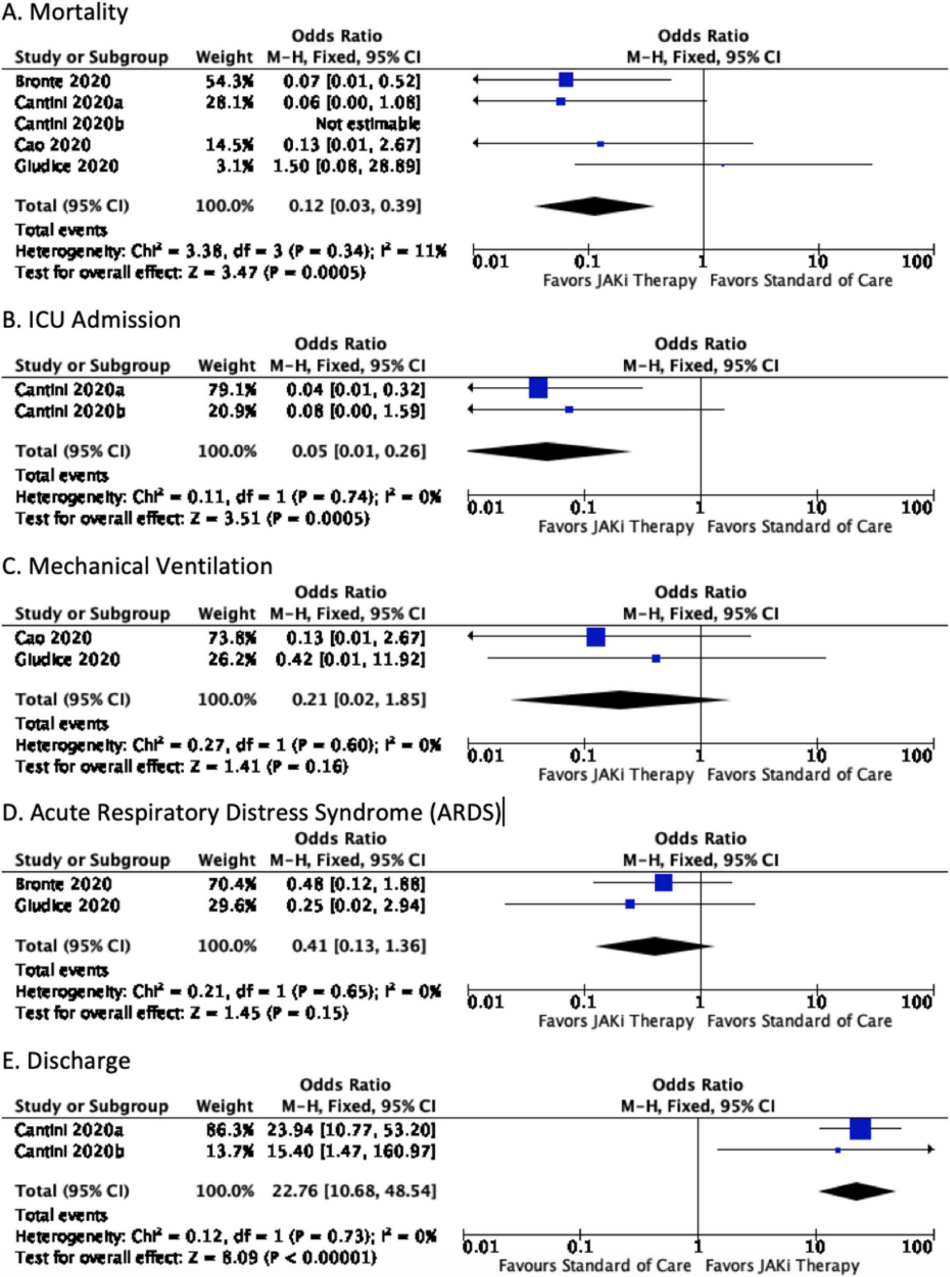
Forest plot of (**a**) Mortality, (**b**) ICU Admission, (**c**) Requirement of Mechanical Ventilation, (**d**) ARDS, and (**e**) Discharge of patients treated with JAK-inhibitor. The fixed effects model was used. JAK-inhibitor treatment group saw significantly reduced odds of mortality and ICU admission, as well as significantly higher odds of discharge, when compared to standard treatment. There was no significant difference between groups in regards to requiring mechanical ventilation, or the development of ARDS. The meta-analysis results are presented on forest plots, with a study’s calculated OR plotted as a black square whose size is proportional to the weight afforded to the study. Bidirectional bars stemming from these black squares correspond to the risk estimate’s 95% CI. Diamonds were used to represent the summary OR; its center aligns with the OR and its width represents the summary 95% CI

A total of five studies investigated the effect of JAK inhibition in a controlled setting (Table 1), enrolling a total of 172 patients who received a JAK-inhibitor and 177 control participants [25–29]. The common parameters that were measured included mortality, ICU admission, requiring mechanical ventilation, incidence of acute respiratory distress syndrome (ARDS), and 14-day discharge. Meta-analysis of the five studies revealed a significantly lower odds of mortality with JAK-inhibitor (OR, 0.12; 95% CI, 0.03–0.39; *p*=0.0005), as compared to standard treatment. The effect size among the different studies demonstrated relatively little heterogeneity (I^2^=11%; Fig. 2a). Pooled analyses of 2 sets of studies revealed that there was no significant association between JAK-inhibitor and COVID-19 patients requiring mechanical ventilation or developing ARDS, respectively (*p*> 0.05; Fig. 2c and d). Both analyses included 27 patients receiving a JAK inhibitor, while the mechanical ventilation and ARDS analyses included 31 and 66 control patients, respectively. Investigation of 125 JAK-inhibitor and 90 control COVID-19 patients found that those treated with JAK-inhibitor, in comparison to those receiving standard treatment, demonstrated 0.05 (95% CI, 0.01–0.26) times the odds of being admitted into the ICU (*p*=0.0005; Fig. 2b). Finally, analysis of 2 studies of 215 patients, 125 of which were treated with a JAK-inhibitor, revealed that those treated with JAK-inhibitor had significantly higher odds than those treated with standard care to be discharged at 2 weeks (OR, 22.76; 95% CI, 10.68–48.54; *p*< 0.00001; Fig. 2e). The analysis examining the relationship between treatment with JAK-inhibition and requiring mechanical ventilation, developing ARDS, ICU admittance, and hospital discharge demonstrated very little heterogeneity (I^2^=0).

### Effect of interferon therapy on clinical outcomes in COVID-19

**Fig. 3:**
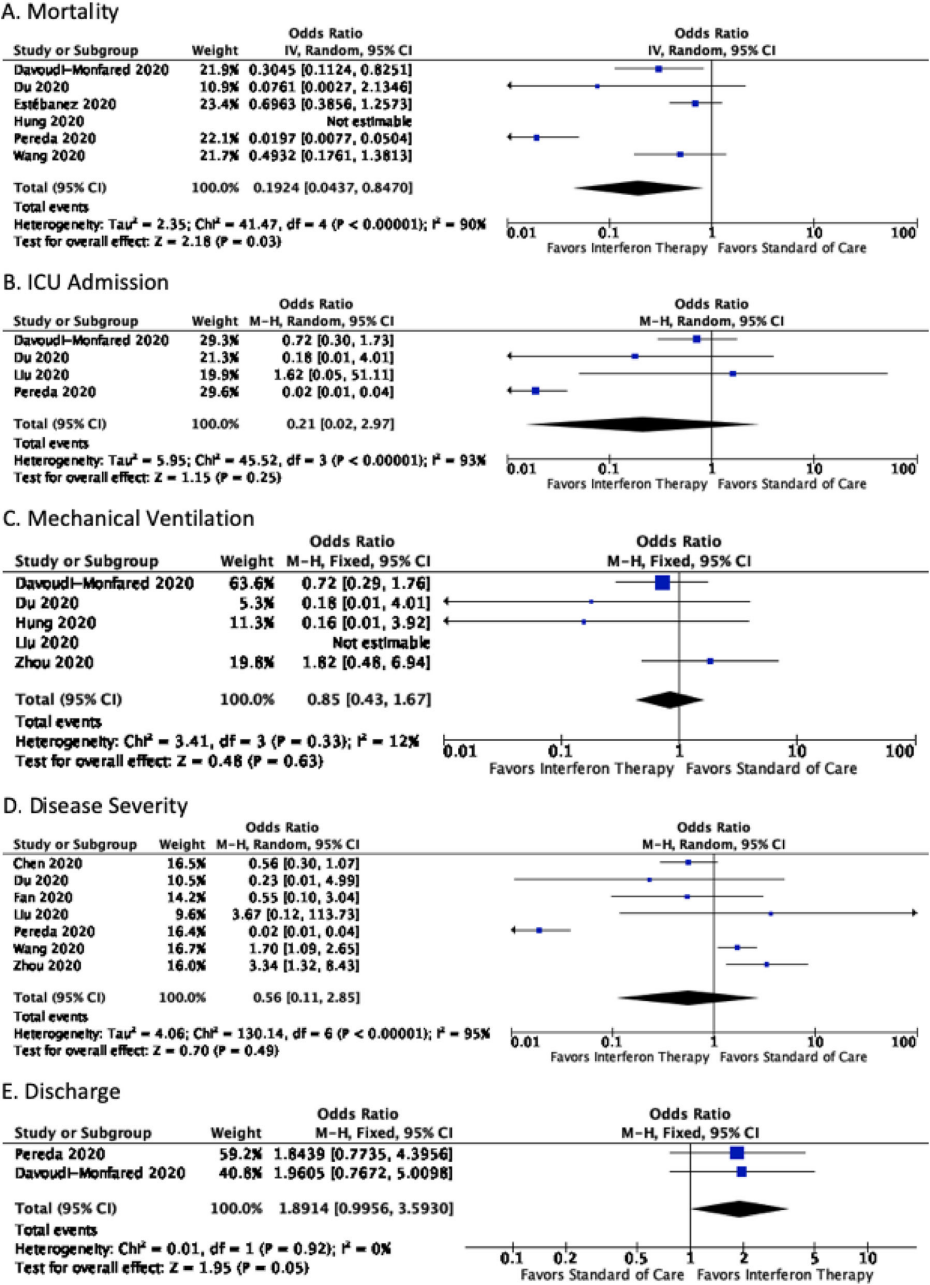
Forest plot of (**a**) Mortality, (**b**) ICU Admission, (**c**) Requirement of Mechanical Ventilation, (**d**) Severe or Critical Disease, and (**e**) Discharge of patients treated with Type I interferon. The fixed effects and random effects model was used dependent on the I^2^ measure of heterogeneity**.** Type I interferon group saw significantly reduced odds of mortality, as well as increased odds of discharge that bordered significance, when compared to standard treatment. There was no significant difference between groups in regards to requiring ICU admission, mechanical ventilation, or the development of severe or critical disease. The meta-analysis results are presented on forest plots, with a study’s calculated OR plotted as a black square whose size is proportional to the weight afforded to the study. Bidirectional bars stemming from these black squares correspond to the risk estimate’s 95% CI. Diamonds were used to represent the summary OR; its center aligns with the OR and its width represents the summary 95% CI

Meta-analysis of 3 sets of studies with 990, 454, and 1480 patients receiving Type I interferon therapy revealed that there were no significant associations between receiving Type I interferon therapy and ICU admittance, requiring mechanical ventilation, or developing a severe or critical case of COVID-19 (*p*> 0.05; Fig. 3b, c, d) compared to the control arm [30–32, 34–39]. The analyses included 97, 167, and 537 control patients, respectively. The data exhibited very high heterogeneity in cases of ICU admittance and disease severity (both I^2^> 90%), but relatively low in the case of mechanical ventilation (I^2^=12%). In the analyses of the 803 and 1415 Type I interferon receiving patients, intervention therapy was respectively associated with higher odds of being discharged (OR, 1.89; 95% CI, 1.00–3.59; *p*=0.05; *N*=895; Fig. 3e), and significantly lower odds of mortality (OR, 0.19; 95% CI, 0.04–0.85); *p*=0.03, *N*=1906; Fig. 3a), when compared to standard of care. The studies included in these analyses enlisted 92 and 491 control patients, respectively. Discharge data exhibited very low heterogeneity (I^2^=0%), while mortality data demonstrated very high heterogeneity (I^2^=90%).

## Discussion

As SARS-CoV-2 continues to infect millions and kill thousands daily, there is an urgent need to find novel therapies that can effectively limit COVID-19 severity. Type I interferon therapy and JAK-inhibitors represent paradoxical approaches to treat COVID-19. While Type I interferon therapy aims to limit the viral replication at the early time points to limit the subsequent disease, JAK-inhibitors aim to limit the overt inflammation that may be detrimental to the host and cause systemic inflammatory response. However, no major randomized clinical trials have been performed to determine their efficacy in limiting the disease severity in COVID-19. Many randomized clinical trials examining the effect of JAK-inhibitors or Type I Interferon therapy for treatment of COVID-19 patients are underway [40, 41].

To our knowledge, this is the first systematic review and meta-analysis to investigate the role of JAK-inhibitors or Type I interferons on clinical outcomes in patients with COVID-19. The results suggest a robust association between JAK-inhibitor and significantly decreased odds of mortality and ICU admission, as well as significantly increased odds for patient discharge within 2 weeks. Furthermore, a significant association between Type I interferon and reduced mortality was also found, in addition to an association with hospital discharge that bordered significance. These results suggest the potential benefit of these therapeutic options for COVID-19.

Although this study presents evidence of JAK-inhibitors and Type I interferon therapies for COVID-19 patients, the evaluated studies included conflicting results: Giudice et al. reported a positive association between JAK-inhibitor therapy and the odds of mortality [29], while the other studies analyzed demonstrated a negative association between JAK-inhibitor intervention and mortality [25, 26, 28]. In addition, two studies consistently demonstrated opposite associations between Type I interferon therapy and clinical outcomes [36, 39], compared to other included studies [30–32, 34, 37]. Heterogeneity among study populations may play a role in the disparate results, as half of these studies were conducted in China, one in Iran, five in Western Europe, and one in Cuba. Other irreconcilable factors that may have influenced patient outcomes included individual study exclusion criterion, as well as the dosage and delivery method of the intervention.

Furthermore, as recent findings have shown that persistent viral presence contributes to disease severity [42], the timing of the administration of both interventions may be of utmost importance. As JAK-inhibitors attenuate JAK signaling and subsequent cytokine release, their administration may best be suited for patients with progressing COVID-19 who have not yet experienced a cytokine storm [43]. By contrast, as Type I interferons induce cellular antiviral states via the JAK/STAT pathway, its administration may be most efficacious early on in disease where the virus is still replicating. While the literature surrounding this is sparse, one study included in this meta-analysis concluded that early administration of interferon-alpha-2b could induce positive outcomes in COVID-19 patients compared to standard treatment, while its late administration was associated with slower recovery [38].

It is important to highlight that this meta-analysis attempted to overcome the challenges posed by studies with insufficient power to detect an effect between JAK-inhibitor or Type I interferon treatment and clinical outcomes, as half of the included studies in this analysis utilized sample sizes less than 100 [25, 27–29, 31, 34, 36]. Nevertheless, despite the broad range of sample sizes and populations, the screening step of our analysis predominantly resulted in low effect size heterogeneity as evidenced by the I^2^ statistics displayed in Fig. 2 and Fig. 3.

This study contained no restrictions regarding study type in the exclusion criteria and, as such, many of the studies included are of retrospective design. Accordingly, baseline characteristics of patients cannot be ignored, especially as factors such as age, gender, and pre-existing comorbidities have been found in meta-analyses to be linked to negative clinical outcomes, including mortality, among COVID-19 patients [44]. One study in particular contained a large disparity in the distribution of chronic conditions across those who received Type I interferon therapy and controls [37]. Furthermore, this meta-analysis included two studies consisting of similar study teams that examined the same association [26, 27], enhancing the likelihood of bias in the same direction in analyses where both of these studies were included.

In addition to heterogeneous outcome reporting, the small sample sizes in peer-reviewed literature would not have provided enough statistical power for meta-analysis if not for the inclusion of articles in pre-print. The inclusion of pre-published work in meta-analyses has long been debated but are currently of utmost importance to push for larger randomized clinical trials during this critical time. Their inclusion may help attenuate artificially extreme effect estimates and counteract publication bias, but may leave the study vulnerable to further bias and misrepresentation [45, 46]. For that reason, all included studies, published and pre-published, were individually reviewed in the evaluation of bias according to randomization status. Risk-of-bias assessments are presents in Supplementary Table 3 and Supplementary Table 4.

## Conclusions

This meta-analysis supports the value of JAK-inhibitors and Type I interferon therapy in combating SARS-CoV-2 infection. This study consolidates existing data and reaffirms the conclusion that, within COVID-19 patients, JAK-inhibitor treatment is significantly associated with positive clinical outcomes in terms of mortality, ICU admission, and discharge, as well as Type I interferon treatment’s association with positive clinical outcomes in regard to mortality and discharge. Although these findings should assist physicians in deciding which antivirals to administer to SARS-CoV-2 infected patients, they also point to a clear need for additional well-designed RCTs examining the relationship of JAK-inhibitors and Type I interferon and clinical outcomes of COVID-19 patients.

## Supplementary Information

**Additional file 1: Supplementary Figures. Supplementary Figure 1.** Funnel plots of JAK-inhibitor treatment for (A) Mortality, (B) ICU Admission, (C) Requirement of Mechanical Ventilation, (D) ARDS, and (E) Discharge. **Supplementary Figure 2**. Funnel plots of Type I interferon treatment for (A) Mortality, (B) ICU Admission, (C) Requirement of Mechanical Ventilation, (D) Severe or Critical Disease, and (E) Discharge. **Supplementary Table 1**. Total outcome data stratified by included study. Supplementary Table 2. Definition of a severe or critical case in included studies for which that measure was analyzed. **Supplementary Table 3**. Risk of Bias (RoB) 2 check list for detection of bias in randomized trials. **Supplementary Table 4**. Newcastle-Ottowa Scale (NOS) tool for risk of bias detection in non-randomized trials. **Supplementary Table 5**. PRISMA-P 2015 checklist: recommended items to address in a systematic review protocol.

## Data Availability

The datasets used and analyzed during the current study are available from the corresponding author on reasonable request.
