# Supplementary material for: JAK-inhibitor and type I interferon ability to produce favorable clinical outcomes in COVID-19 patients: a systematic review and meta-analysis
Source: BMC Infect Dis. 2021 Jan 11;21:47. doi: 10.1186/s12879-020-05730-z (PMC7797881; doi:10.1186/s12879-020-05730-z)
Supplement: Supplementary file 1 — Additional file 1: Supplementary Figures. Supplementary Figure 1. Funnel plots of JAK-inhibitor treatment for (A) Mortality, (B) ICU Admission, (C) Requirement of Mechanical Ventilation, (D) ARDS, and (E) Discharge. Supplementary Figure 2. Funnel plots of Type I interferon treatment for (A) Mortality, (B) ICU Admission, (C) Requirement of Mechanical Ventilation, (D) Severe or Critical Disease, and (E) Discharge. Supplementary Table 1. Total outcome data stratified by included study. Supplementary Table 2. Definition of a severe or critical case in included studies for which that measure was analyzed. Supplementary Table 3. Risk of Bias (RoB) 2 check list for detection of bias in randomized trials. Supplementary Table 4. Newcastle-Ottowa Scale (NOS) tool for risk of bias detection in non-randomized trials. Supplementary Table 5. PRISMA-P 2015 checklist: recommended items to address in a systematic review protocol. [file 12879_2020_5730_MOESM1_ESM.docx]

**Supplementary Figure 1. Funnel plots of JAK-inhibitor treatment for (A) Mortality, (B) ICU Admission, (C) Requirement of Mechanical Ventilation, (D) ARDS, and (E) Discharge**

**Supplementary Figure 2. Funnel plots of Type I interferon treatment for (A) Mortality, (B) ICU Admission, (C) Requirement of Mechanical Ventilation, (D) Severe or Critical Disease, and (E) Discharge**

95% CI lines were not presented when the random effects model was used.

**Supplementary Table 1. Total outcome data stratified by included study**

Studies presented in alphabetical order by treatment group.

**Supplementary Table 2. Definition of a severe or critical case in included studies for which that measure was analyzed.**

Studies presented in alphabetical order.

**Supplementary Table 3. Risk of Bias (RoB) 2 check list for detection of bias in randomized trials.**

Avenues of bias considered: Risk of bias arising from the randomization process, Risk of bias due to deviations from the intended interventions (effect of assignment to intervention), Risk of bias due to deviations from the intended interventions (effect of adhering to intervention), Risk of bias due to missing outcome data, Risk of bias due to measurement of the outcome, Risk of bias in selection of the reported result. (N=No; PN=Probably No; Y=Yes; PY= Probably Yes; NI= Not Indicated; NA= Not Applicable)

**Supplementary Table 4. Newcastle-Ottowa Scale (NOS) tool for risk of bias detection in non-randomized trials.**

Study characteristics examined: (1) representativeness of exposed cohort, (2) selection of nonexposed cohort, (3) exposure assessment, (4) outcome of interest not present at the start of the study, (5) comparability of cohorts, (6) outcome assessment, (7) adequacy of length of time before follow-up, and (8) adequacy of follow-up of cohorts

**Supplementary Table 5. PRISMA-P 2015 checklist: recommended items to address in a systematic review protocol.**

**Supplementary Figure 1. Funnel plots of JAK-inhibitor treatment for (A) Mortality, (B) ICU Admission, (C) Requirement of Mechanical Ventilation, (D) ARDS, and (E) Discharge**


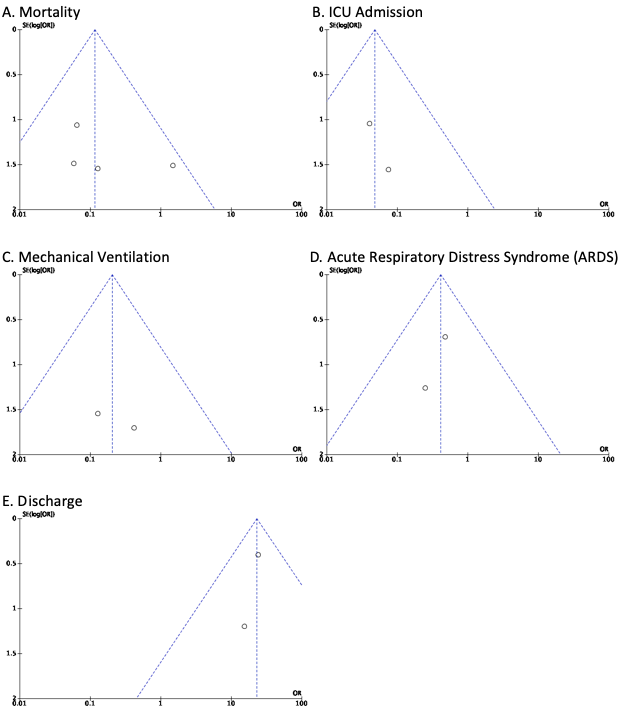


**Supplementary Figure 2. Funnel plots of Type I interferon treatment for (A) Mortality, (B) ICU Admission, (C) Requirement of Mechanical Ventilation, (D) Severe or Critical Disease, and (E) Discharge.**

**
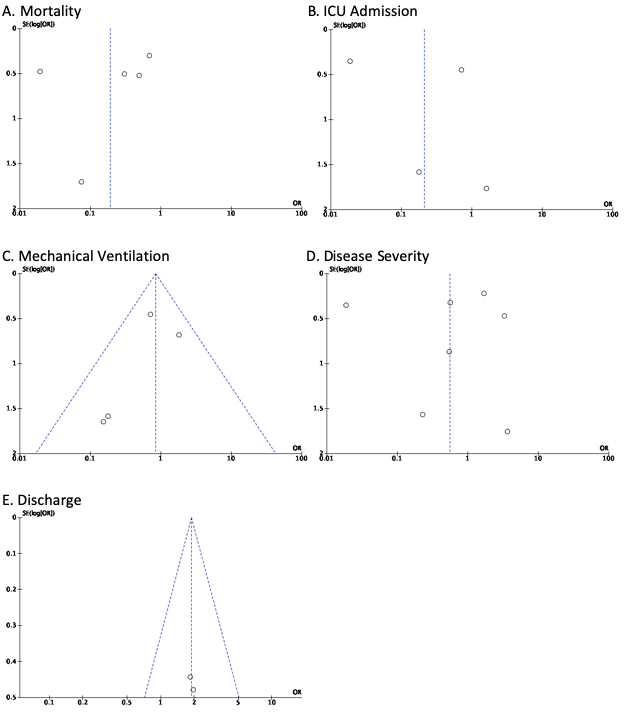
**

**Supplementary Table 1. Total outcome data stratified by included study**

| First Author, Year | Total Deaths | Cases where IMV was required | Cases admitted to ICU | Total Severe + Critical Cases | ARDS | Discharge |
| --- | --- | --- | --- | --- | --- | --- |
| Bronte 2020 | 26 | * | * | * | 18 | * |
| Cantini 2020a | 5 | * | 15 | * | * | 98 |
| Cantini 2020b | 0 | * | 4 | * | * | 8 |
| Cao 2020 | 3 | 3 | * | * | * | * |
| Giudice 2020 | 2 | 1 | * | * | 5 | * |
| Chen 2020 | * | * | * | 50 | * | * |
| Davoudi-Monfared 2020 | 25 | 32 | 36 | * | * | 54 |
| Du 2020 | 1 | 3 | 3 | 4 | * | * |
| Estébanez 2020 | 63 | * | * | * | * | * |
| Fan 2020 | * | * | * | 8 | * | * |
| Hung 2020 | 0 | 1 | * | * | * | * |
| Liu 2020 | * | 0 | 3 | 5 | * | * |
| Pereda 2020 | 24 | * | 82 | 82 | * | 151 |
| Wang 2020 | 16 | * | * | 111 | * | * |
| Zhou 2020 | * | 12 | * | 35 | * | * |

* Data was not reported across control and treatment group or not clear

**Supplementary Table 2. Definition of a severe or critical case in included studies for which that measure was analyzed.**

| First Author, Year | Definition of Severe + Critical Case |
| --- | --- |
| Chen 2020 | One of the following: (1) respiratory distress, respiratory rate ≥ 30 times/min; (2) fingertip oxygen saturation ≤ 93% in resting state; (3) arterial oxygen partial pressure/fraction of inspired oxygen ≤300 mmHg. |
| Davoudi-Monfared 2020 | (1) hypoxemia (need for noninvasive or invasive respiratory support to provide capillary oxygen saturation above 90%) (2) Hypotension (systolic blood pressure less than 90 mmHg or vasopressor requirement) (3) renal failure secondary to COVID-19 (according to KDIGO definition) [18] (4) neurologic disorder secondary to COVID-19 (decrease of 2 or more scores in Glasgow Coma Scale) (5) thrombocytopenia secondary to COVID-19 (platelet count less than 150000 /mm3) (6) severe gastrointestinal symptoms secondary to COVID-19 (vomiting/diarrhea that caused at least mild dehydration). |
| Du 2020 | One of the following criteria: (1) shortness of breath with increased respiratory rate (RR) except for the influence of fever and crying (RR 60 breaths per minute for those younger than 2 60 months, RR 50 breaths per minute for those aged between 2 and 12 months, RR 40 breaths per minute 61 for those aged between 1 and 5 years, and RR 30 breaths per minute for those older than 5 years); (2) oxygen saturation 92% at rest; (3) hypoxia with accessory respiration (groaning, flaring of nares, three concave sign), cyanosis, and intermittent apnea; (4) disturbance of consciousness with somnolence and convulsions; (5) food refusal or feeding difficulty, with signs of dehydration; (6) high-resolution CT showing bilateral or multi-lobe involvement, with rapid aggressiveness or pleural effusion. |
| Fan 2020 | One of the following criteria: (1) Respiratory distress (≧30 breaths/ min); (2) Oxygen saturation≤93% at rest; (3) Arterial partial pressure of oxygen (PaO2)/ fraction of inspired oxygen (FiO2)≦ 300mmHg (l mmHg=0.133kPa). In high-altitude areas (at an altitude of over 1,000 meters above the sea level), PaO2/ FiO2 shall be corrected by the following formula: PaO2/ FiO2 x[Atmospheric pressure (mmHg)/760] Cases with chest imaging that shows obvious lesion progression within 24-48 hours >50% shall be managed as severe cases. Child cases meeting any of the following criteria: (1) Tachypnea (RR ≥ 60 breaths/min for infants aged below 2 months; RR ≥ 50 BPM for infants aged 2-12 months; RR ≥ 40 BPM for children aged 1-5 years, and RR ≥ 30 BPM for children above 5 years old) independent of fever and crying; (2) Oxygen saturation ≤ 92% on finger pulse oximeter taken at rest; (3) Labored breathing (moaning, nasal fluttering, and infrasternal, supraclavicular and intercostal retraction), cyanosis, and intermittent apnea; (4) Lethargy and convulsion; (5) Difficulty feeding and signs of dehydration. |
| Wang 2020 |  |
| Liu 2020 | One of the following: resting oxygen saturation less than 93% or arterial partial oxygen pressure <60 mm Hg or respiratory rate more than 30 breaths per minute. |
| Pereda 2020 | Artificial Ventilation, ARDS |
| Zhou 2020 | ^a^ |

^a^ Information was not presented

**Supplementary Table 3. RoB 2 check list for detection of bias in randomized trials.**

|  |  | Cao 2020 | Davoudi-Monfared 220 | Giudice 2020 | Hung 2020 |
| --- | --- | --- | --- | --- | --- |
| Bias due to randomization | |  |  |  |  |
| 1.1 | Was the allocation sequence random? | Y | Y | Y | Y |
| 1.2 | Was the allocation sequence concealed until participants were enrolled and assigned to interventions? | PY | NI | NI | N |
| 1.3 | Did baseline difference between intervention groups suggest a problem with the randomization process? | N | PN | NI | N |
|  | **Risk-of-bias judgement** | Low | Some Concerns | Some Concerns | High |
|  | Optional: What is the predicted direction of bias arising from the randomization process? |  |  |  |  |
| Bias due to deviation from assignment to intervention | |  |  |  |  |
| 2.1 | Were participants aware of their assigned intervention during the trial? | N | NI | NI | Y |
| 2.2 | Were carers and people delivering the interventions aware of the participants’ assigned intervention during the trial? | Y | NI | NI | Y |
| 2.3 | **If Y/PY/NI to 2.1 or 2.2:** Were there deviations from the intended intervention balanced between groups | N | PN | N | PN |
| 2.4 | **If Y/PY to 2.3:** Were these deviations likely to have affected the outcome? | NA | NA | NA | NA |
| 2.5 | **If Y/PY/NI to 2.4:** Were these deviations from intended intervention balanced between groups? | NA | NA | NA | NA |
| 2.6 | Was an appropriate analysis used to estimate the effect of assignment to intervention? | Y | PY | Y | Y |
| 2.7 | **If N/PN/NI to 2.6:** Was there potential for a substantial impact (on the result) of the failure to analyze participants in the group to which they were randomized? | NA | NA | NA | NA |
|  | **Risk-of-bias judgement** | Low | Low | Low | Low |
|  | Optional: What is the predicted direction of bias arising from the randomization process? |  |  |  |  |
| Bias due to deviation from adherence to intervention | |  |  |  |  |
| 2.1 | Were participants aware of their assigned intervention during the trial? | N | NI | NI | Y |
| 2.2 | Were carers and people delivering the interventions aware of participants' assigned intervention during the trial? | Y | NI | NI | Y |
| 2.3 | **If Y/PY/NI to 2.1 or 2.2:** Were important non-protocol interventions balanced across intervention groups? | NI | NI | PN | NI |
| 2.4 | Were there failures in implementing the intervention that could have affected the outcome? | PN | PN | PN | PN |
| 2.5 | Was there non-adherence to the assigned intervention regimen that could have affected participants’ outcomes? | NI | NI | NI | NI |
| 2.6 | **If N/PN/NI to 2.3, or Y/PY/NI to 2.4 or 2.5:** Was an appropriate analysis used to estimate the effect of adhering to the intervention? | PY | PY | PY | Y |
|  | **Risk-of-bias judgement** | Some Concerns | Some Concerns | Some Concerns | Some Concerns |
|  | Optional: What is the predicted direction of bias arising from the randomization process? |  |  |  |  |
| Bias due to missing outcome data | |  |  |  |  |
| 3.1 | Were data for this outcome available for all, or nearly all, participants randomized? | Y | Y | N | Y |
| 3.2 | **If N/PN/NI to 3.1:** Is there evidence that the result was not biased by missing outcome data? | NA | NA | PY | NA |
| 3.3 | **If N/PN to 3.2:** Could missingness in the outcome depend on its true value? | NA | NA | NA | NA |
| 3.4 | **If Y/PY/NI to 3.3:** Is it likely that missingness in the outcome depended on its true value? | NA | NA | NA | NA |
|  | **Risk-of-bias judgement** | Low | Low | Low | Low |
|  | Optional: What is the predicted direction of bias arising from the randomization process? |  |  |  |  |
| Bias due to outcome measurement | |  |  |  |  |
| 4.1 | Was the method of measuring the outcome inappropriate? | N | N | N | N |
| 4.2 | Could measurement or ascertainment of the outcome have differed between intervention groups? | PN | PN | PN | N |
| 4.3 | **If N/PN/NI to 4.1 and 4.2:** Were outcome assessors aware of the intervention received by study participants? | Y | NI | NI | Y |
| 4.4 | **If Y/PY/NI to 4.3:** Could assessment of the outcome have been influenced by knowledge of intervention received? | PY | PY | NI | PN |
| 4.5 | **If Y/PY/NI to 4.4:** Is it likely that assessment of the outcome was influenced by knowledge of intervention received? | PN | NI | PN | NA |
|  | **Risk-of-bias judgement** | Low | High | Some Concerns | Low |
|  | Optional: What is the predicted direction of bias arising from the randomization process? |  |  |  |  |
| Bias due to selection of reported result | |  |  |  |  |
| 5.1 | Were the data that produced this result analyzed in accordance with a pre-specified analysis plan that was finalized before unblinded outcome data were available for analysis? | Y | Y | Y | Y |
| 5.2 | Is the numerical result being assessed likely to have been selected, on the basis of the results, from multiple eligible outcome measurements (e.g. scales, definitions, time points) within the outcome domain? | N | N | N | N |
| 5.3 | Is the numerical result being assessed likely to have been selected, on the basis of the results, from multiple eligible analyses of the data? | N | N | N | N |
|  | **Risk-of-bias judgement** | Low | Low | Low | Low |
|  | Optional: What is the predicted direction of bias arising from the randomization process? |  |  |  |  |
| Overall Risk-of-Bias | |  |  |  |  |
|  | **Risk-of-bias judgement** | Low | Some Concerns | Low | Low |
|  | Optional: What is the predicted direction of bias arising from the randomization process? |  |  |  |  |

(N=No; PN=Probably No; Y=Yes; PY= Probably Yes; NI= Not Indicated; NA= Not Applicable)

**Supplementary Table 4. Newcastle-Ottowa Scale (NOS) tool for risk of bias detection in non-randomized trials.**

| Study | Outcome | Selection domain  (maximum 4 stars) | Comparability domain  (maximum 2 stars) | Outcome domain  (maximum 3 stars) |
| --- | --- | --- | --- | --- |
| Bronte 2020 | Mortality | 4 | 0 | 2 |
| Bronte 2020 | ARDS^a^ | 3 | 0 | 2 |
| Cantini 2020a | Mortality | 4 | 0 | 2 |
| Cantini 2020a | ICU Admission | 4 | 0 | 3 |
| Cantini 2020a | Discharge | 4 | 0 | 3 |
| Cantini 2020b | Mortality | 4 | 0 | 2 |
| Cantini 2020b | ICU Admission | 4 | 0 | 3 |
| Cantini 2020b | Discharge | 4 | 0 | 3 |
| Chen 2020 | Disease Severity | 3 | 0 | 3 |
| Du 2020 | Mortality | 4 | 0 | 3 |
| Du 2020 | ICU Admission | 3 | 0 | 3 |
| Du 2020 | Mechanical Ventilation | 3 | 0 | 3 |
| Estébanez 2020 | Mortality | 4 | 0 | 3 |
| Fan 2020 | Disease Severity | 3 | 0 | 3 |
| Liu 2020 | ICU Admission | 4 | 0 | 2 |
| Liu 2020 | Mechanical Ventilation | 4 | 0 | 2 |
| Liu 2020 | Disease Severity | 3 | 0 | 2 |
| Pereda 2020 | Mortality | 4 | 0 | 1 |
| Pereda 2020 | ICU Admission | 4 | 0 | 1 |
| Pereda 2020 | Disease Severity | 3 | 0 | 1 |
| Pereda 2020 | Discharge | 4 | 0 | 1 |
| Wang 2020 | Mortality | 4 | 0 | 3 |
| Wang 2020 | Disease Severity | 3 | 0 | 3 |
| Zhou 2020 | Mechanical Ventilation | 4 | 0 | 3 |
| Zhou 2020 | Disease Severity | 4 | 0 | 3 |

^a^ ARDS = Acute Respiratory Distress Syndrome

**Supplementary Table 5. PRISMA-P (Preferred Reporting Items for Systematic review and Meta-Analysis Protocols) 2015 checklist: recommended items to address in a systematic review protocol***

| Section and topic | Item No | Checklist item | Page |
| --- | --- | --- | --- |
| ADMINISTRATIVE INFORMATION | | |  |
| Title: |  |  |  |
| Identification | 1a | Identify the report as a protocol of a systematic review | 1 |
| Update | 1b | If the protocol is for an update of a previous systematic review, identify as such | 1 |
| Registration | 2 | If registered, provide the name of the registry (such as PROSPERO) and registration number | 1 |
| Authors: |  |  |  |
| Contact | 3a | Provide name, institutional affiliation, e-mail address of all protocol authors; provide physical mailing address of corresponding author | 1 |
| Contributions | 3b | Describe contributions of protocol authors and identify the guarantor of the review | 14,15 |
| Amendments | 4 | If the protocol represents an amendment of a previously completed or published protocol, identify as such and list changes; otherwise, state plan for documenting important protocol amendments | 1 |
| Support: |  |  |  |
| Sources | 5a | Indicate sources of financial or other support for the review | 14 |
| Sponsor | 5b | Provide name for the review funder and/or sponsor | 14 |
| Role of sponsor or funder | 5c | Describe roles of funder(s), sponsor(s), and/or institution(s), if any, in developing the protocol | 14 |
| INTRODUCTION | | |  |
| Rationale | 6 | Describe the rationale for the review in the context of what is already known | 5,6 |
| Objectives | 7 | Provide an explicit statement of the question(s) the review will address with reference to participants, interventions, comparators, and outcomes (PICO) | 5 |
| METHODS | | |  |
| Eligibility criteria | 8 | Specify the study characteristics (such as PICO, study design, setting, time frame) and report characteristics (such as years considered, language, publication status) to be used as criteria for eligibility for the review | 7,8 |
| Information sources | 9 | Describe all intended information sources (such as electronic databases, contact with study authors, trial registers or other grey literature sources) with planned dates of coverage | 7 |
| Search strategy | 10 | Present draft of search strategy to be used for at least one electronic database, including planned limits, such that it could be repeated | 7 |
| Study records: |  |  |  |
| Data management | 11a | Describe the mechanism(s) that will be used to manage records and data throughout the review | 7 |
| Selection process | 11b | State the process that will be used for selecting studies (such as two independent reviewers) through each phase of the review (that is, screening, eligibility and inclusion in meta-analysis) | 7 |
| Data collection process | 11c | Describe planned method of extracting data from reports (such as piloting forms, done independently, in duplicate), any processes for obtaining and confirming data from investigators | 7 |
| Data items | 12 | List and define all variables for which data will be sought (such as PICO items, funding sources), any pre-planned data assumptions and simplifications | 7,8 |
| Outcomes and prioritization | 13 | List and define all outcomes for which data will be sought, including prioritization of main and additional outcomes, with rationale | 8 |
| Risk of bias in individual studies | 14 | Describe anticipated methods for assessing risk of bias of individual studies, including whether this will be done at the outcome or study level, or both; state how this information will be used in data synthesis | 13 |
| Data synthesis | 15a | Describe criteria under which study data will be quantitatively synthesised | 8 |
|  | 15b | If data are appropriate for quantitative synthesis, describe planned summary measures, methods of handling data and methods of combining data from studies, including any planned exploration of consistency (such as I^2^, Kendall’s τ) | 9,10 |
|  | 15c | Describe any proposed additional analyses (such as sensitivity or subgroup analyses, meta-regression) | 13 |
|  | 15d | If quantitative synthesis is not appropriate, describe the type of summary planned | 1 |
| Meta-bias(es) | 16 | Specify any planned assessment of meta-bias(es) (such as publication bias across studies, selective reporting within studies) | 13 |
| Confidence in cumulative evidence | 17 | Describe how the strength of the body of evidence will be assessed (such as GRADE) | 13 |

*** It is strongly recommended that this checklist be read in conjunction with the PRISMA-P Explanation and Elaboration (cite when available) for important clarification on the items. Amendments to a review protocol should be tracked and dated. The copyright for PRISMA-P (including checklist) is held by the PRISMA-P Group and is distributed under a Creative Commons Attribution Licence 4.0.**

*From: Shamseer L, Moher D, Clarke M, Ghersi D, Liberati A, Petticrew M, Shekelle P, Stewart L, PRISMA-P Group. Preferred reporting items for systematic review and meta-analysis protocols (PRISMA-P) 2015: elaboration and explanation. BMJ. 2015 Jan 2;349(jan02 1):g7647.*
